# A shift from drought to extreme rainfall drives a stable landslide to catastrophic failure

**DOI:** 10.1038/s41598-018-38300-0

**Published:** 2019-02-07

**Authors:** Alexander L. Handwerger, Mong-Han Huang, Eric Jameson Fielding, Adam M. Booth, Roland Bürgmann

**Affiliations:** 1 Jet Propulsion Laboratory, California Institute of Technology, Pasadena, CA 91109 USA; 2 Department of Geology, University of Maryland, College Park, MD 20742 USA; 3 Department of Geology, Portland State University, Portland, OR 97207 USA; 4 Berkeley Seismological Laboratory, University of California, Berkeley, CA 94720 USA; 5 Department of Earth and Planetary Science, University of California, Berkeley, CA 94720 USA

## Abstract

The addition of water on or below the earth’s surface generates changes in stress that can trigger both stable and unstable sliding of landslides and faults. While these sliding behaviours are well-described by commonly used mechanical models developed from laboratory testing (e.g., critical-state soil mechanics and rate-and-state friction), less is known about the field-scale environmental conditions or kinematic behaviours that occur during the transition from stable to unstable sliding. Here we use radar interferometry (InSAR) and a simple 1D hydrological model to characterize 8 years of stable sliding of the Mud Creek landslide, California, USA, prior to its rapid acceleration and catastrophic failure on May 20, 2017. Our results suggest a large increase in pore-fluid pressure occurred during a shift from historic drought to record rainfall that triggered a large increase in velocity and drove slip localization, overcoming the stabilizing mechanisms that had previously inhibited landslide acceleration. Given the predicted increase in precipitation extremes with a warming climate, we expect it to become more common for landslides to transition from stable to unstable motion, and therefore a better assessment of this destabilization process is required to prevent loss of life and infrastructure.

## Introduction

Stress and fluid-pressure perturbations from changes in infiltrating precipitation and snowmelt^1–7^, seasonal water storage^8^, dehydration reactions^9^, and wastewater and fluid-injection^10–12^ can trigger diverse sliding behaviours of both landslides and faults, including stable sliding (e.g., slow-moving landslides or aseismic fault slip) and unstable sliding (e.g., fast-moving landslides or earthquakes). These various sliding behaviours are described by the laboratory-based critical-state soil mechanics^3–5^ and rate-and-state friction^10–15^ models. In general, stable sliding should occur if the frictional resistance increases during sliding and unstable sliding should occur if the frictional resistance decreases during sliding. These changes in the frictional resistance are controlled by material properties^10–16^ and/or from shear-induced changes in pore-fluid pressure^3–5,17^. Although these mechanical models can be used to describe various sliding behaviours, less is known about the field-scale environmental conditions or kinematic behaviours that occur during the transition from stable to unstable sliding. In this manuscript, we present new remote sensing observations that show the behaviour of a landslide that transitioned from stable to unstable sliding.

Ongoing natural and man-made climate shifts (i.e., global warming) in response to increasing concentrations of greenhouse gases are predicted to increase the frequency and magnitude of geohazards such as landslides^18^. Recent studies have investigated the role of climate change on slope stability using historical records of temperature, precipitation, and landslide events with future predictions from downscaled Global Circulation Models^18–20^. These studies have found that the occurrence or activity of landslides is expected to increase in areas where the frequency and magnitude of precipitation is predicted to increase and decrease in areas where the frequency and magnitude of precipitation is predicted to decrease. However, interactions between ongoing climate shifts and landslide behaviour are difficult to assess due to uncertainties in both climate and landslide models. Documenting the behaviour of landslides in response to ongoing climate shifts is essential for improving our understanding of the mechanisms that control their velocity and our ability to forecast catastrophic failure.

The California Coast Ranges are an ideal natural laboratory to investigate how stress and fluid pressure changes govern the stable and unstable sliding of landslides. Landslides are pervasive in these mountains due to active uplift, mechanically weak lithologies, and high seasonal precipitation^1,21–27^. Slow-moving landslides mainly occur within areas of the Coast Ranges that are comprised of the Jurassic-Cretaceous Franciscan mélange, an uplifted accretionary prism complex consisting of a clayey granular matrix with highly sheared sandstone, siltstone, meta-sandstone, shale, serpentinite, and blueschist^22,28^. More specifically, the landslides occur in weak, clay-rich regolith^29^. The California Coast Ranges have a Mediterranean Climate with average precipitation values of ~1.0 m/yr in the Central Coast Ranges and ~1.5 m/yr in the Northern Coast Ranges, 80% of which falls between October and May. However, precipitation can vary significantly from year to year due to changes in the frequency and strength of atmospheric rivers^30,31^, El Niño-Southern Oscillation events^26,32^, and over longer time scales with the Pacific Decadal Oscillation^33^. For instance, between 2015 and 2017 there was a transition from historic drought to one of the wettest years on record. Predictions from the Community Earth System Model Large Ensemble (CESM) indicate that over the next century annual mean precipitation could increase up to 8 cm in Central California and 14 cm in Northern California^34^. CESM simulations also predict a 100 to 200% increase in cumulative seasonal precipitation and a 25 to 100% increase in the frequency of dry-to-wet year extremes that may be similar to the recent precipitation shifts in California^35^.

**Figure 1:**
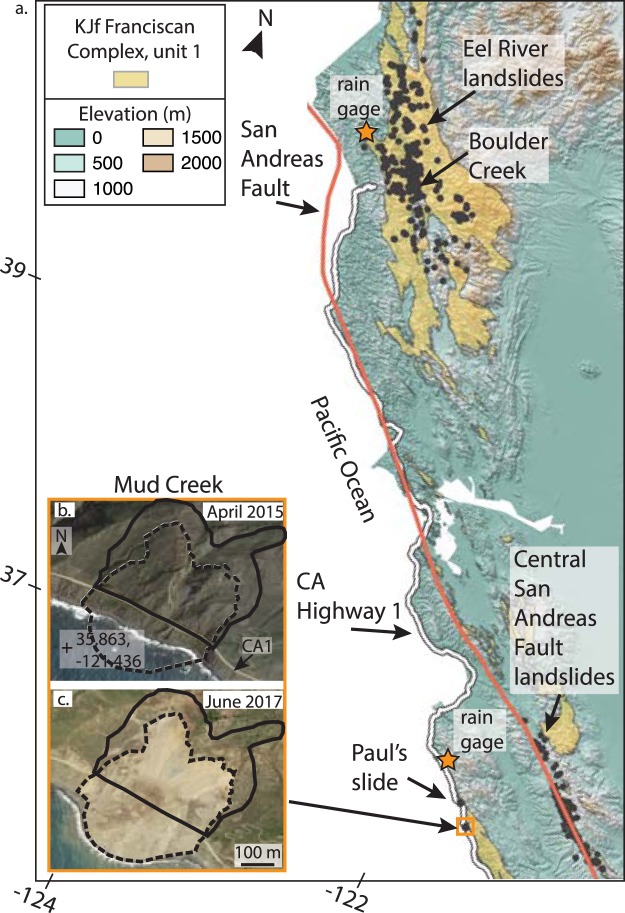
Northern and Central California Coast Ranges. (**a**) Elevation and Franciscan Complex lithologic unit 1 and San Andreas fault^28^ draped over a hillshade of the topography. Black polygons show mapped inventories of slow-moving landslides^22–25^. Map data: Geologic map from USGS^28^ (https://mrdata.usgs.gov/geology/state), digital elevation models from TanDEM-X. TanDEM-X data used is under copyright by the DLR. (**b**,**c**) Google Earth images (Map data: SIO, NOAA, U.S. Navy, NGA, GEBCO; Image; Landsat/Copernicus) of the Mud Creek landslide before and after catastrophic failure. Solid black and dashed black polygons shows pre- and post-catastrophic failure landslide boundaries. We mapped the pre-catastrophic failure boundaries using InSAR, Google Earth, and a digital elevation model. We mapped the post-catastrophic failure landslide boundaries using Google Earth. Software: QGIS Geographic Information System. Open Source Geospatial Foundation Project. http://qgis.osgeo.org.

More than 650 slow-moving landslides have been identified and mapped in the California Coast Ranges in recent years, many of which have been active for decades or longer, and almost none of which have failed catastrophically^22–25^. Yet, the Mud Creek landslide, which is located within the Franciscan mélange (Fig. 1; Extended Data Fig. 1), suddenly failed catastrophically on a dry day (May 20, 2017) following a prolonged period of heavy rainfall and destroyed California State Highway 1 (CA1)^36,37^. There was no loss of human life caused by this landslide, due in part to quick decision making by the California Department of Transportation (Caltrans) that resulted in closing of the road between May 2017 and July 2018, however the landslide resulted in a cost of $54 million in repairs^38^. Since its construction in the 1920’s, CA1 has been closed more than 50 times due to landslides^39^, and based on the predictions of future climate change^30,31,34,35^, landslide frequency and activity is likely to increase over the next century.

In this study, we investigate how a shift from drought to extreme rainfall can affect the behaviour of landslides. We measure the time-dependent deformation of three landslides from satellite and airborne InSAR and model precipitation-induced changes in pore-fluid pressure using a simple homogenous 1D diffusion model. We use our remote sensing observations and hydrologic models to explore interactions between precipitation and landslide kinematics. We then discuss our results in the context of the critical-state soil mechanics and rate and state friction fault mechanics frameworks.

## Results

**Figure 2:**
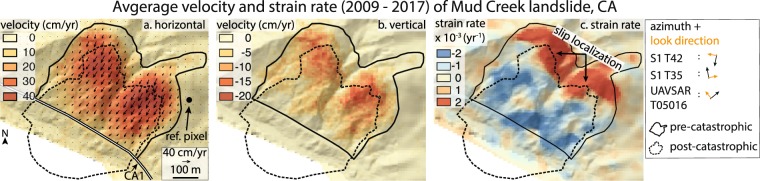
Velocity and strain rate maps of Mud Creek landslide. (**a**) Average horizontal velocity between February 2009 and May 2017 with velocity vectors draped over hillshade of the topography. CA1 shown with black and white line. Reference pixel corresponds to a stable area outside the landslide. (**b**) Average vertical velocity with negative values indicating downward motion. (**c**) Strain rate map showing upslope horizontal extension (positive values) and downslope contraction (negative values). The azimuth and look direction of the Sentinel-1A/B and UAVSAR SAR instruments are shown with black and orange arrows in the legend. Map data: digital elevation models from USGS (https://earthexplorer.usgs.gov/) and TanDEM-X. TanDEM-X data used is under copyright by the DLR. All rights reserved, used with permission. Software: QGIS Geographic Information System. Open Source Geospatial Foundation Project. http://qgis.osgeo.org.

We use satellite and airborne InSAR data to measure the deformation history of the Mud Creek landslide between 2009 and 2017 and two other landslides (Paul’s Slide and Boulder Creek landslide) in the California Coast Ranges between 2015 and 2017 (Methods). This time period is particularly well-suited for studying the effects of rainfall on landslide behaviour because it spanned the final year (2015) of a historic drought and one of the wettest years on record (2017). The Mud Creek landslide displayed a minimum of 8 years of stable sliding prior to its catastrophic failure (Extended Data Fig. 2). Average velocities near the fastest moving part of the landslide between 2009 and 2017 were 0.43 m/yr towards the south, 0.24 m/yr towards the west, and 0.17 m/yr vertically downward (Fig. 2 and Extended Data Fig. 3). Velocities were highest near the catastrophic failure headscarp and approached zero (i.e. no slip) where the landslide intersected the road. The landslide consisted of two lobes separated by a channel incised 20 to 30 meters into the landslide body. The two lobes moved uniformly in time suggesting they were connected by a single sliding surface at depth (Extended Data Fig. 4 and Supplementary Movie 1). Interestingly, the area of the landslide that eventually failed catastrophically was smaller than the total deforming body and was located further downslope. The headscarp of the catastrophic failure formed in the zone of extension, near the transition from extension to compression (Fig. 2c). The area of the landslide was 0.23 km^2^ prior to catastrophic failure, but the exposed failure scar has a minimum area of 0.079 km^2^. This threefold difference in area indicates that slip localized along a different lateral (and possibly basal) sliding surface.

**Figure 3:**
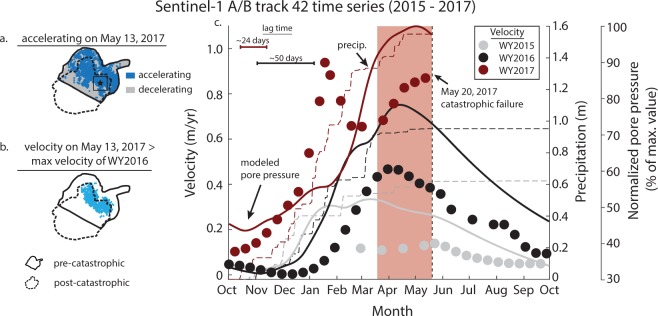
Velocity time series of Mud Creek landslide. (**a**) Parts of the landslide that were accelerating on May 13, 2017. (**b**) Parts of the landslide with velocities greater than the maximum velocities during WY2016. (**c**) Downslope velocity, precipitation, and modelled pore-fluid pressure time series during WY2015-WY2017 condensed into a single calendar year for a representative area (averaged over 60 × 60 m, shown by black box and star in (**a**); Extended Data Fig. 5). The normalized pore-fluid pressure is defined as the pore-fluid pressure divided by the maximum value over the study period. Lag time corresponds to the time between the onset of precipitation and onset of acceleration. Red rectangle highlights the divergence from the characteristic seasonal velocity pattern.

Our time series analysis between 2015 and 2017 shows that Mud Creek displayed nearly continuous downslope motion with seasonal velocity changes that scaled with precipitation (Fig. 3, Extended Data Fig. 4, and Extended Data Fig. 5). Due to differences in the timing and magnitude of rainfall over the study period, the landslide displayed differences in displacement and velocity each year. The lowest velocities occurred during the 2015 drought. Although the data does not span the full 2015 water year (Oct. 1, 2014 – Sep. 30, 2015, referred to as “WY2015”), we observed low velocities (values close to zero) within the late Winter/early Spring, which typically corresponds to periods of elevated velocities in coastal California landslides^1,7,40^. Additionally, our results agree with a recent study that showed that landslide velocities reached a historic low between 2012 and 2015 in the Northern California Coast Ranges^41^. There was an increase in velocity during WY2016, which appears to correspond directly to the increased rainfall. The landslide accelerated within 1.5 months of the onset of precipitation, reached its maximum slip rate between April and May, and decelerated throughout the dry season. Finally, the extreme rainfall of WY2017 resulted in an even larger and more rapid increase in velocity. During WY2017, the onset of seasonal precipitation was rapid and more intense. Accelerated slip began within 1 month of the onset of rainfall, and the landslide reached maximum velocities in January 2017, 3 to 4 months earlier than in previous years. Although Mud Creek began to decelerate during the wet season, which suggests that stabilizing mechanisms such as slip- or rate-strengthening friction or a decrease in pore-fluid pressure from dilatancy and drainage may have been preventing runaway acceleration^4,11^, a series of late season rain storms triggered a second period of acceleration that caused the landslide to maintain high velocities until its ultimate collapse. We find that most of the landslide was accelerating at the time of the last satellite acquisition prior to catastrophic failure (May 13, 2017), and the velocities within the headscarp region were higher than they had been during the previous two years (Fig. 3, Extended Data Fig. 4, and Extended Data Fig. 5). Our InSAR time series generally agrees with displacements recorded by multi-temporal lidar and structure-from-motion data collected by the USGS^36^. Their data shows minor displacement between 2010 and 2016 and an increase in displacement between 2016 and 2017. Their final two data acquisitions between March 8, 2017 and May 19, 2017 show much larger displacements when compared to the previous 6 years, reveal the development of the failure headscarp, and show a smaller surficial slide that blocked part of Highway 1.

Increases in slip each year tracked the transition from historic drought to extreme rainfall and imply that the transient (i.e. seasonal) pore-fluid pressure increase that causes a reduction in frictional strength and drives accelerated slip varied drastically between WY2015 and WY2017. We used a simple, homogenous 1D diffusion model to simulate the changes in subsurface pore-fluid pressure induced by precipitation events at Mud Creek (Methods). Because we have no direct measurement of pore-fluid pressure, diffusivity, or thickness from these landslides, we explored relative changes in the normalized pore-fluid pressure, defined as the pore-fluid pressure divided by the maximum value over the study period. Our results show that the minimum pore-fluid pressures occurred during the drought of WY2015 and approximately tripled by WY2017 (Fig. 3 and Extended Data Fig. 4). Assuming that the groundwater levels change by a few meters from the dry to wet season, we can infer that the pore-fluid pressure changes are on the order of kilopascals^1,2,4^. Our first-order model is able to describe the overall velocity patterns of Mud Creek during WY2015 and WY2016. While our model predicts a large pore-fluid pressure rise in WY2017, it does not address the more complex velocity patterns observed with our InSAR data.

**Figure 4:**
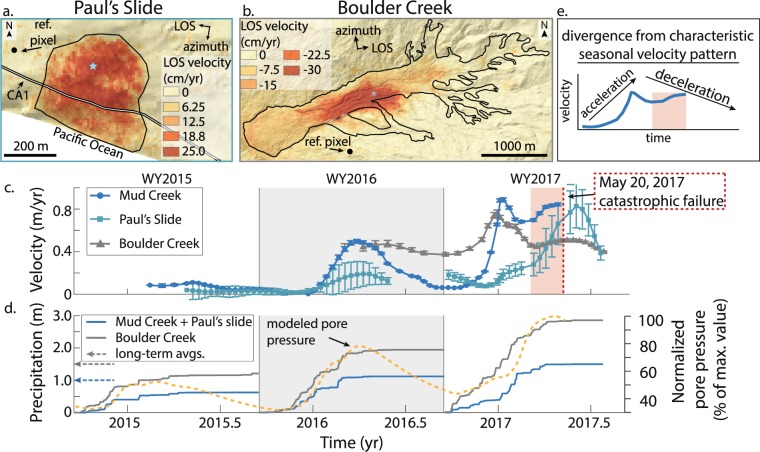
Sentinel-1 A/B velocity time series comparison. (**a**,**b**) Average LOS velocity for Paul’s Slide and Boulder Creek landslide draped over a hillshade of the topography. Negative values correspond to motion towards the satellite line-of-sight (LOS). CA1 shown with black and white line. Reference pixel corresponds to a stable area outside the landslide. Black polygons show landslide boundaries. (**c**) Velocity time series projected onto the downslope direction for representative areas of each landslide (blue and grey stars in **a** and **b**). Error bars show the uncertainty estimates calculated using a Jackknife test (Methods). Due to low coherence in many of the interferograms, we do not have a complete time series for Boulder Creek or Paul’s Slide. (**d**) Precipitation time series for the Central and Northern California Coast Ranges and modelled pore-fluid pressure for Mud Creek. Rain gage locations shown in Fig. 1. (**e**) WY2017 velocity time series for Mud Creek highlighting the divergence from the characteristic seasonal velocity pattern of slow-moving landslides in the California Coast Ranges (highlighted by red rectangle). Map data: Digital elevation models from OpenTopgraphy (http://www.opentopography.org) and TanDEM-X. TanDEM-X data used is under copyright by the DLR. All rights reserved, used with permission. Software: QGIS Geographic Information System. Open Source Geospatial Foundation Project. http://qgis.osgeo.org.

We compared the velocity time series of Mud Creek to the Boulder Creek landslide and nearby Paul’s Slide (Fig. 1), two landslides that have displayed stable sliding for decades^22,36,42^. These landslides occur within similar lithology and in similar climates to Mud Creek (Extended Data Fig. 1). Paul’s Slide displayed low velocities during the drought of WY2015, and all of the landslides displayed a similar velocity pattern consisting of gradual and smooth acceleration followed by deceleration during WY2016 (Fig. 4), although Boulder Creek maintained a moderate velocity leading into WY2017. During WY2017, however, the three landslides exhibited contrasting behaviours. Boulder Creek and Mud Creek accelerated and reached their peak velocities at about the same time, but Boulder Creek continued to decelerate while Mud Creek accelerated catastrophically. Interestingly, Paul’s Slide displayed seasonal motion in WY2017 that was similar to its motion in WY2016, but attained a much higher velocity after a 6-month lag. We note that the surface deformation of Paul’s Slide may be influenced by the ongoing roadwork and hillslope grading by Caltrans^36^. Lastly, due to a lack of ground-based measurements of landslide thickness and soil diffusivity, we did not model the pore-fluid pressure changes for each landslide individually. Differences in thickness and diffusivity result in differences in the timing and magnitude of the predicted pore-fluid pressures. Yet, the Mud Creek landslide model captures the first-order velocity patterns of Paul’s Slide and Boulder Creek during WY2016 and Paul’s Slide in WY2017, which suggests the landslides experience similar first-order hydrologic changes (Fig. 4).

## Discussion

The long-term stable sliding displayed by these landslides has been well-documented at numerous landslides in the California Coast Ranges^21,22^. In fact, Boulder Creek has been moving in this fashion for over 70 years^22^. Yet, after ≥8 years of slow sliding, Mud Creek rapidly transitioned to catastrophic failure, indicating that there was a change in its stability conditions. While Mud Creek does not appear to display creep-to-failure behaviour^43,44^, which is manifested as a hyperbolic increase in velocity towards catastrophic failure, we do observe a clear divergence from the typical velocity patterns displayed by slow-moving landslides in the California Coast Ranges (Fig. 4). These precursory behavioural differences may correspond to a change in the landslide stability regime. However, it is also possible that we failed to observe creep-to-failure behaviour if it occurred over the last several days prior to catastrophic failure or if the landslide displacements significantly exceeded the InSAR deformation thresholds (Methods).

During stable sliding, a balance must exist between transient weakening from pore-fluid pressure increase due to precipitation that drives increased slip rates and (i) transient strengthening from a decrease in pore-fluid pressure due to dilation and drainage^3–5,45^ and/or (ii) intrinsic slip- or rate-strengthening material properties^10–15^. Additional stabilization may result from an increase in a critical nucleation size relative to the slip patch size. This is an important length scale because rate-and-state friction theory predicts unstable sliding should only occur if (i) the frictional strength decreases during slip events and (ii) the slip patch exceeds the critical nucleation size defined by h^*^ = *Gd*_*c*_/*σ’*(*b*-*a*), where *G* is the shear modulus, *σ*′ is the effective normal stress (normal stress minus pore-fluid pressure), *b* and *a* are friction parameters, and *d*_c_ is the characteristic slip distance for the evolution of frictional contacts^10–15^. Importantly, this length scale suggests that that the increased pore-fluid pressure that drives acceleration also acts to increase the critical nucleation size, and therefore predicts that an increase in pore-fluid pressure should drive increased slip but should not trigger runaway instability. Yet, landslides and faults can both display dynamic sliding due to increased pore-fluid pressures^2,6,11^.

One way to transition to unstable sliding is for the slip patch size to increase and become larger than the critical nucleation size^15^. However, we observed that the Mud Creek landslide decreased in size during catastrophic failure (Fig. 2). Another way a landslide can transition from stable to unstable sliding is to switch from slip- or rate-strengthening behaviour to slip- or rate-weakening behaviour^12,16^. This can occur due to increased displacement, which can cause a transition from shear-induced dilatancy to compaction and can rapidly increase pore-fluid pressures^2,4,17^ or from large increases in pore-fluid pressures due to precipitation that overcome frictional strengthening mechanisms^11^.

Catastrophic failure of the Mud Creek landslide is most likely due to frictional weakening that was initiated by large increases in pore-fluid pressure. These large stress changes resulted from extreme rainfall that followed a period of historic drought and may have been enhanced by increased pore-fluid pressures from shear-induced compaction and longitudinal compression of the upslope material due to differential slip^3,4,17^. Our pore-fluid pressure model does not account for multi-dimensional pore-fluid pressure changes^46,47^ or for mechanical-hydrologic feedbacks such as shear-induced dilatancy or compaction^4,45^, however this simple model has been widely used to describe the first-order changes in pore-fluid pressure that drive acceleration of similar deep-seated landslides around the world^1,2,48,49^ and indicates that the Mud Creek landslide experienced unusually high pore-fluid pressures prior to catastrophic failure. Furthermore, we hypothesize that the preceding drought and extension near the headscarp likely increased rapid infiltration pathways such as tension cracks that can facilitate strong pore-fluid pressure changes^46^. Our results also show that slip localization occurred just prior to catastrophic failure (Fig. 2c; Supplementary Movie 1). Slip localization occurs in landslide and fault materials that weaken with slip and has been found to coincide with runaway instability in laboratory experiments^11,50^.

Despite the behavioural change of the Mud Creek landslide, Boulder Creek and Paul’s slide, among many landslides in the area, were able to adjust to the high pore-fluid pressures and maintain stable sliding. Differences in the stress at the landslide base, drainage networks, slip surface thickness and porosity, and landslide geometry (Extended Data Table 1) are among the factors that may explain these behavioural differences. For example, the Mud Creek landslide has a mean slope angle of 32 degrees, which is steeper than many of the slow-moving landslides in the California Coast Ranges (mean slope angles ~19 degrees)^22^, and therefore had a higher gravitational driving stress. Additional measurements, including direct measurements of porosity and pore-fluid pressure in the sliding surface are required to provide more insight into the mechanisms that control these various slip modes.

Our findings agree with recent laboratory investigations that found that large pore-fluid pressure increases from wastewater and fluid injection can trigger earthquakes along otherwise stable faults and provide additional field-scale evidence that large stress changes can lead to a transition in stability regimes^11,12^. Yet, these findings seemingly contradict predictions of commonly used mechanical models (e.g., rate-and-state friction), which currently do not describe how large increases in pore-fluid pressure can trigger dynamic instability on stable sliding surfaces and suggests that modifications that include descriptions of critical-state mechanics may be required to better characterize these sliding behaviours.

Because precipitation and precipitation extremes (i.e. dry to wet years) are predicted to rise in California over the next several decades^30,31,34,35^, we hypothesize that there may be an increase in the occurrence and activity of landslides in California. And furthermore, there may be an increase in the transition from stable to unstable landslide motion if these precipitation extremes generate sufficiently high pore-fluid pressures. Our results highlight how state-of-the-art InSAR data can be used to quantify the deformation history of landslides prior to catastrophic failure, especially in areas where timely installation of on-the-ground instrumentation is not feasible. We suggest that changes in the long-term characteristic deformation patterns may provide a way to identify changes in landslide stability regimes and to forecast imminent failure, especially on landslide-prone transport corridors such as California Highway 1.

## Methods

### InSAR processing and model correction to fix unwrapping errors

We perform interferometric processing of synthetic aperture radar (InSAR) images acquired by the Copernicus Sentinel-1 A/B satellites and the airborne NASA Uninhabited Aerial Vehicle Synthetic Aperture Radar (UAVSAR). The InSAR data are processed with the InSAR Scientific Computing Environment (ISCE) software package developed at JPL/Caltech and Stanford^51^. We use a 12 m pixel spacing digital elevation model from the DLR TanDEM-X to remove topographic contributions to the phase and to geocode the interferograms. We apply a standard power spectral filter with a value of 0.5 to reduce phase noise^52^. The Sentinel-1A/B satellites acquire C-band (5.6 cm radar wavelength) images with a minimum repeat acquisition interval of 6 days (12 days for each satellite, with a 6-day interval between Sentinel-1A and Sentinel-1B. We use ascending (satellite moving north looking east) and descending (satellite moving south, looking west) data collected in the Interferometric Wide swath mode, which has a 250 km swath with 14 m azimuth and 5 m range pixel resolution. Data is processed with 2 looks in range and 1 look in azimuth. We process interferograms with time spans ranging from 6 to 48 days. Prior to our data analysis we removed all poor quality interferograms that have low coherence. Poor quality interferograms were identified by manual inspection of each individual interferogram. The interferograms used in our analysis are listed in Supplementary Tables 1 to 5. For the Mud Creek landslide, we use ascending Track 35 and descending Track 42. A total of 63 descending images are used to produce 178 interferograms between March 2015 and May 2017 (Supplementary Table 1). We also process 5 interferogram pairs between June 2017 and August 2017, which is after the catastrophic failure (Extended Data Fig. 6). The post-catastrophic failure interferograms record both natural and anthropogenic (i.e. road construction) deformation and we cannot easily interpret the displacements. A total of 25 ascending images are used to produce 71 interferograms between August 2016 and May 2017 (Supplementary Table 2). For Paul’s Slide, we use data from descending Track 42 to produce 159 interferograms (Supplementary Table 3). For the Boulder Creek landslide, we use 40 images from ascending Track 35 acquired between January 2016 and October 2017 to produce 124 interferograms (Supplementary Table 4). We also produce 13 interferograms from the L-band (24 cm radar wavelength) UAVSAR Track 05016 (aircraft moving northeast at heading 50 degrees and looking northwest) system at the Mud Creek landslide (Supplementary Table 5 and Extended Data Fig. 2). UAVSAR has a resolution of 1.67 m in range and 0.6 m in azimuth and we processed the data using 3 range looks and 8 azimuth looks, respectively. The data spans from 2009 to 2017 and is typically acquired twice per year, however there is only one acquisition between 2015 and 2017.

We process the InSAR data using conventional 2-pass techniques. However, given the large deformation of these landslides, conventional InSAR techniques sometimes fail because InSAR requires the displacement between adjacent pixels to be less than half the radar wavelength for accurate phase unwrapping^25^. To overcome issues associated with these deformation thresholds, we used a scalable model to correct for large deformation at the Mud Creek (Extended Data Fig. 7) and Boulder Creek landslides prior to unwrapping. We did not make these corrections for Paul’s Slide because we were not able to construct a reliable deformation model. Since ISCE does not yet allow for a deformation model to be input into the processing scheme, we perform the model correction using MATLAB. This is done in 5 steps: (1) we construct a deformation model using the temporal average of interferograms that do not contain phase jumps from unwrapping errors due to deformation thresholds (Supplementary Data Tables 1, 2, 4). We apply a mask to the landslides and set values to zero outside of the landslide area. (2) We scale the model deformation to match the duration of the interferogram being processed. (3) We remove the deformation model from the filtered wrapped interferogram using

1 $${I}_{r}=\exp \,(1j\ast {I}_{wrap})\exp \,(-\,1j\ast {I}_{model}),$$

where *I*_*r*_ is the filtered residual wrapped interferograms (i.e. model removed), *I*_*wrap*_ is the filtered original interferograms, *j* is the basic imaginary unit, and *I*_*model*_ is the unwrapped deformation model. (4) We unwrap the residual interferogram, *I*_*r*_ using the Statistical-Cost, Network-Flow Algorithm for Phase Unwrapping (SNAPHU)^53^. (5) Finally, we add the deformation model back to the unwrapped residual interferogram. Each interferogram is inspected to ensure no additional errors are introduced. Refs^7,25^ successfully implemented this technique to process interferograms that were previously limited by deformation thresholds at slow-moving landslides in the California Coast Ranges.

To construct time series inversions for the Sentinel-1 A/B interferograms, we use the Generic InSAR Analysis Toolbox (GIAnT)^54^. We use the Small Baseline Subset (SBAS) method^55,56^ and apply a coherence threshold of 0.2. This coherence filter removes low quality pixels from the time series analysis (Extended Data Fig. 4). Then we analyse the time series data in MATLAB. To highlight the overall patterns of motion and improve the signal-to-noise ratio, we smooth the displacement time series with a loess filter (locally weighted linear regression) with a window size of ~180 days (Extended Data Fig. 8). We use a 180-day window size because it is approximately the wavelength of the seasonal precipitation cycle. However, this smoothing may possibly introduce a temporal shift to the acceleration patterns. The raw data is presented in Extended Data Fig. 6 which highlights some potential differences in the timing and landslide behaviours. Finally, we calculate the velocity time series using the central difference approximation in a numerical finite difference calculation.

For our time series analysis of Mud Creek, we examine data from the descending Sentinel-1 Track 42, which had an acquisition 7 days before the catastrophic failure (May 13, 2017; Supplementary Table 1). We also analyse data from the ascending Sentinel-1 Track 35, which had an acquisition 1 day before the catastrophic failure (May 19, 2017; Supplementary Table 2). Because the landslide had moved several meters on May 19, 2017^36^, which is well beyond the deformation threshold of InSAR^25^, we were not able to reliably measure the last interval. We compared the velocity time series from the ascending and descending tracks and find good agreement of the temporal pattern of the velocity (Extended Data Fig. 9). Finally, we estimate time series uncertainties by performing a Jackknife test. We find uncertainties on the order of millimetres to centimetres per year (Fig. 4). For the representative velocity time series of Mud Creek shown in Fig. 4, the mean uncertainty is 6.602 ± 6.586 mm/yr (±1std). For the representative velocity time series of Paul’s Slide and Boulder Creek, the mean uncertainty is 74.30 ± 60.19 mm/yr (±1std) and 16.70 ± 9.872 mm/yr (±1std), respectively.

### Projecting line-of-sight velocity onto the downslope direction

To provide better constraints on the true landslide velocity when 3D inversions are not possible, we back-projected the line-of-sight (LOS) motion onto the downslope direction^7,23,32^. This requires a vector transformation to account for the geometry of the radar instrument and the downslope direction of the landslide. We define the unit look vector for the radar ${\hat{l}}_{i}$at each pixel *i* as

2 $${\hat{l}}_{i}=\,\sin \,{\xi }_{i}\,\sin \,{\theta }_{i}\hat{n}-\,\cos \,{\xi }_{i}\,\sin \,{\theta }_{i}\hat{e}+\,\cos \,{\theta }_{i}\hat{z},$$

where ${\xi }_{i}$ is the azimuth flight direction (in degrees) of the radar platform in the horizontal plane from North, ${\theta }_{i}$ is the look angle from nadir, and $\hat{n}$ corresponds to the North component, $\hat{e}$ to the East component, and $\hat{z}$ to the vertical component. We define the unit vector for the downslope sliding direction of the landslide ${\hat{d}}_{ls,i}$ at each pixel as

3 $${\hat{d}}_{ls,i}=\,\sin ({\zeta }_{i})\sin \,({\alpha }_{i})\hat{n}-\,\cos ({\zeta }_{i})\,\sin \,({\alpha }_{i})\hat{e}+\,\cos \,({\alpha }_{i})\hat{z},$$

where ${\zeta }_{i}$ is the orientation of the downslope direction in the horizontal plane from North, and ${\alpha }_{i}$ is the hillslope angle. These values are found in the radar parameter files or measured from the TanDEM-X digital elevation models. We smooth the hillslope angle measured from the DEM using a 2D median filter with a window size of 180 m, which better characterizes the overall downslope direction. Note that while these equations describe the LOS and downslope direction, often the InSAR vectors must be rotated by 90 degrees to account for the conventions of the specific InSAR processing software. Finally, the downslope velocity at each pixel is

4 $${V}_{i}={o}_{i}{({\hat{l}}_{i}\cdot {\hat{d}}_{ls,i})}^{-1},$$

where ${V}_{i}$ is the downslope projected velocity and ${o}_{i}$ is the LOS velocity.

### 3D velocity inversion from InSAR

We combine data with 3 LOS measurements (i.e. UAVSAR and Sentinel-1 A/B ascending and descending) to invert for the full 3D velocity field at the Mud Creek landslide (Extended Data Fig. 3). Due to differences in acquisition dates between UAVSAR and Sentinel-1 A/B we used temporally averaged velocity values for each dataset and assume this approximates the long-term average velocity of the landslide. We define the velocity vector, $\overrightarrow{v}$ as the linear combination of basis vectors ${\hat{m}}_{i}$^57^

5 $$\vec{v}=\sum _{i=1}^{3}\,{v}_{i}{\hat{m}}_{i}=\sum _{i=1}^{3}\,\langle \vec{v},{\hat{m}}_{i}\rangle \,{\hat{m}}_{i}.$$

The LOS measurements (${o}_{i}$) are comprised of the true velocity vectors projected onto the LOS direction ($\widehat{{l}_{i}}$) and can be formulated as a least squares problem

6 $$\vec{o}\,=A\vec{v},$$

where $\overrightarrow{o}$ (*N* x 1) is the LOS measurement (*N* = 3), *A* (N x 3) is the model matrix with LOS vectors projected onto the basis vectors such that

7 $$A=[\begin{array}{ccc}\langle {\hat{l}}_{1},{\hat{m}}_{1}\rangle  & \langle {\hat{l}}_{1},{\hat{m}}_{2}\rangle  & \langle {\hat{l}}_{1},{\hat{m}}_{3}\rangle \\ \vdots  & \vdots  & \vdots \\ \langle {\hat{l}}_{N},{\hat{m}}_{1}\rangle  & \langle {\hat{l}}_{N},{\hat{m}}_{2}\rangle  & \langle {\hat{l}}_{N},{\hat{m}}_{3}\rangle \end{array}],$$

and

8 $$\vec{v}={({A}^{t}QA)}^{-1}{A}^{t}Q\vec{o},$$

where *Q* is the estimated covariance matrix. We calculate *Q* from the pixel correlation using the Cramer-Rao bounds^58^

9 $$Q=\frac{\lambda }{4\pi }\sqrt{\frac{1-{\gamma }^{2}}{2{\gamma }^{2}}},$$

where *γ* is the pixel coherence and *λ* is the radar wavelength.

### Pore-fluid pressure diffusion model for landslides

Simple, homogenous 1D diffusion models have been widely used in landslide studies to describe precipitation-induced changes in pore-fluid pressure^1,2,4,48,49^. Our primary modelling goal is to characterize the first-order hydrologic response within the landslide body that drives landslide motion. We model the vertical diffusion of pore-fluid pressure from the ground surface to the basal sliding surface as^1,2,4,48,49^,

10 $$\frac{\partial P}{\partial t}\,=D\frac{{\partial }^{2}P}{\partial {z}^{2}},$$

where *z* is depth from the ground surface, *D* is the effective hydraulic diffusivity of the soil or rock, and *t* is time. We use the precipitation rate time series *R*(*t*) to define the surface boundary condition

11 $$P(t,z=0)=rR(t),$$

where *P*(*t*, *z* = 0) is the ground surface pore-fluid pressure, and *r* is an empirically calibrated infiltration factor that must account for hydraulic properties (e.g., hydraulic conductivity, infiltration capacity) and scales the measured precipitation rate to a pressure value. Alternatively, the downward water flux can be specified at the ground surface, and that boundary condition provides essentially the same result^1,59^. A solution for equation (11) with a transient surface boundary condition is defined as^15,40,59^

12 $$P(z,t)={\int }_{0}^{t}\frac{z}{\sqrt{4\pi D{(t-s)}^{3}}}{e}^{\frac{-{z}^{2}}{4D(t-s)}}P(s,z=0)ds,$$

where *s* is a constant. We solve this equation in the Fourier domain by

13 $$F(t) \sim \frac{z}{\sqrt{4\pi D{(t-s)}^{3}}}{e}^{\frac{-{z}^{2}}{4D(t-s)}},$$

14 $$G(s) \sim  {\mathcal F} [P(t,z=0)],$$

15 $$P(t,z) \sim { {\mathcal F} }^{-1}(F\cdot G),$$

where ${\mathcal F}$ is the Fourier transform.

We run the model to steady-state by repeating the WY2015 rainfall data for 50 years prior to the start of the study period. Since we have no direct measurement of pore-fluid pressure, soil diffusivity, or landslide thickness, we explore relative changes in the normalized pore-fluid pressure. We use an arbitrary infiltration factor *r*, since we normalize the model results. Our model allows us to explore the timing and relative strength of the pore-fluid pressure changes. In order to model true pore-fluid pressure values the infiltration factor *r* needs to be constrained by hydraulic properties or fit to pore-fluid pressure data from landslide boreholes. However, assuming that the groundwater levels change by a few meters from the dry to wet season, we can infer that the pore-fluid pressure changes are on the order of kilopascals^1,2,4,48^. Importantly, our model does not account for multi-dimensional pore-fluid pressure changes^46,47^, shear-induced changes in pore-fluid pressure within the slip surface^4,11,45^, or for spatial or temporal changes in diffusivity^1,48^ or landslide thickness^25^, which may vary significantly within a single landslide.

Equation (15) provides the transient pore-fluid pressure changes located at depth *z* below the ground surface. By varying the landslide thickness or hydraulic diffusivity, which scales the strength and timing of the pore-fluid pressure changes, we can calculate a range of pore-fluid pressure values and better constrain the model parameters (Extended Data Fig. 10). Based on estimates of the Mud Creek landslide volume^36^ and the measured planform area (Extended Data Table 1), we find the average landslide thickness was approximately 20 meters (i.e. volume/area). We solve for the best fit soil diffusivity by minimizing the Root Mean Square Error (RMSE) misfit between the normalized modelled pore-fluid pressure and normalized velocity time series during the WY2016 (Extended Data Fig. 10). We fit the diffusivity during WY2016 (rather than the full time period) because it is the only complete water year of velocity data^42^. For a thickness of 20 m, our best fit soil diffusivity is 1.18 × 10^−5^ m^2^/s, which is within the measured values for a slow-moving landslide in the California Coast Ranges^1^. We find good agreement between the modelled pore-fluid pressure and measured velocity changes (Fig. 3 and Extended Data Fig. 4 and Extended Data Fig. 10).

### Strain rate calculations

The infinitesimal 2D strain rate tensor (${\dot{\varepsilon }}_{m,n}$) is defined as

16 $${\dot{\varepsilon }}_{m,n}=\frac{1}{2}({u}_{m,n}+{u}_{n,m}),$$

where *m* is the east component and *n* is the north component, and ${u}_{m,n}$ is the velocity gradient in the *x*-direction

17 $${u}_{m,n}=\frac{\partial {v}_{m}}{\partial {x}_{n}}.$$

For this, we assume zero vertical strain. The principal strain rate values and orientations are essentially the eigenvalues and eigenvectors of the strain tensor, and the dilatational strain is the sum of these eigenvalues. A 2D strain rate tensor can be calculated with at least 3 measurements in space. Because our InSAR measurements provide high spatial coverage (~150 m^2^/pixel), even small data noise can lead to a high variation in the strain rate tensor. For example, 1 mm of measurement error in 30 m distance contributes to ~33 micro-strain rate. We therefore calculate the strain rate tensor of one grid point by considering a range of InSAR pixels. We use 30 m grid points and estimate the strain rate based on the 2D horizontal mean velocities of Mud Creek between 2009 and 2017, neglecting the vertical component. Since we assume no discontinuity in space and no vertical strain, we can use a plane-strain mass continuity equation

18 $${U}_{m}={a}_{1}+{a}_{2}{u}_{m,m}+{a}_{3}{u}_{m,n};m\ne n,$$

to estimate the terms for each grid point

19 $$\frac{\partial {v}_{m}}{\partial {x}_{m}},\frac{\partial {v}_{n}}{\partial {x}_{n}},\frac{\partial {v}_{m}}{\partial {x}_{n}},$$

where *a*_1_, *a*_2_, and *a*_3_ are fit parameters. Figure 2c shows that the upslope area of Mud Creek is extending (positive values) and the downslope area is contracting. The headscarp of the catastrophic failure formed in the extensional zone. It is possible that longitudinal compression of the upslope material further enhanced the increase in pore-fluid pressure that eventually caused catastrophic failure.

### Code availability

All computer codes used in this work is available from the authors upon reasonable request.

## Supplementary information

Supplemenary Movie 1

Extended Data

Supplementary Table 1.

Supplementary Table 2.

Supplementary Table 3.

Supplementary Table 4

Supplementary Table 5

## Data Availability

Contains modified Copernicus Sentinel data 2015–2017, processed by ESA. The InSAR original data used in this paper are freely available and provided by Copernicus and NASA. Sentinel-1 data can be downloaded from the Alaska Satellite Facility (ASF, https://www.asf.alaska.edu) and from the Copernicus Science Hub. UAVSAR data used in this study can be downloaded from https://uavsar.jpl.nasa.gov/. The California geologic map is provided by the USGS (https://mrdata.usgs.gov/geology/state). Lidar digital elevation models were downloaded at USGS Explorer (https://earthexplorer.usgs.gov/) and OpenTopography (http://www.opentopography.org). OpenTopography lidar data acquisition and processing completed by the National Center for Airborne Laser Mapping (NCALM - http://www.ncalm.org). NCALM funding provided by NSF’s Division of Earth Sciences, Instrumentation and Facilities Program. EAR-1043051. Rainfall data for the Big Sur and Redway stations was acquired by NOAA and provided by the California Climate Data Archive (CalClim, https://calclim.dri.edu). Optical images of Mud Creek landslide in Fig. 1 are from Google Earth (Map data: SIO, NOAA, U.S. Navy, NGA, GEBCO; Image; Landsat/Copernicus; https://www.google.com/earth/).
