# Supplementary material for: A shift from drought to extreme rainfall drives a stable landslide to catastrophic failure
Source: Sci Rep. 2019 Feb 7;9:1569. doi: 10.1038/s41598-018-38300-0 (PMC6367458; doi:10.1038/s41598-018-38300-0)
Supplement: Supplementary file 2 — Extended Data [file 41598_2018_38300_MOESM2_ESM.pdf]

Extended Data and Supplementary Information for

A shift from drought to extreme rainfall drives a stable landslide to catastrophic failure

Alexander L. Handwerger*[1], Mong-Han Huang[2], Eric J. Fielding[1], Adam M. Booth[3], Roland Bürgmann[4,5]

[1]Jet Propulsion Laboratory, California Institute of Technology, Pasadena, CA 91109, USA.

[2]Department of Geology, University of Maryland, College Park, MD 20742, USA.

[3]Department of Geology, Portland State University, Portland, OR 97207, USA.

[4]Berkeley Seismological Laboratory, University of California, Berkeley, CA 94720, USA.

[5]Department of Earth and Planetary Science, University of California, Berkeley, CA 94720, USA.

*Correspondence to: alexander.handwerger@jpl.nasa.gov.

© 2018 California Institute of Technology. Government sponsorship acknowledged.

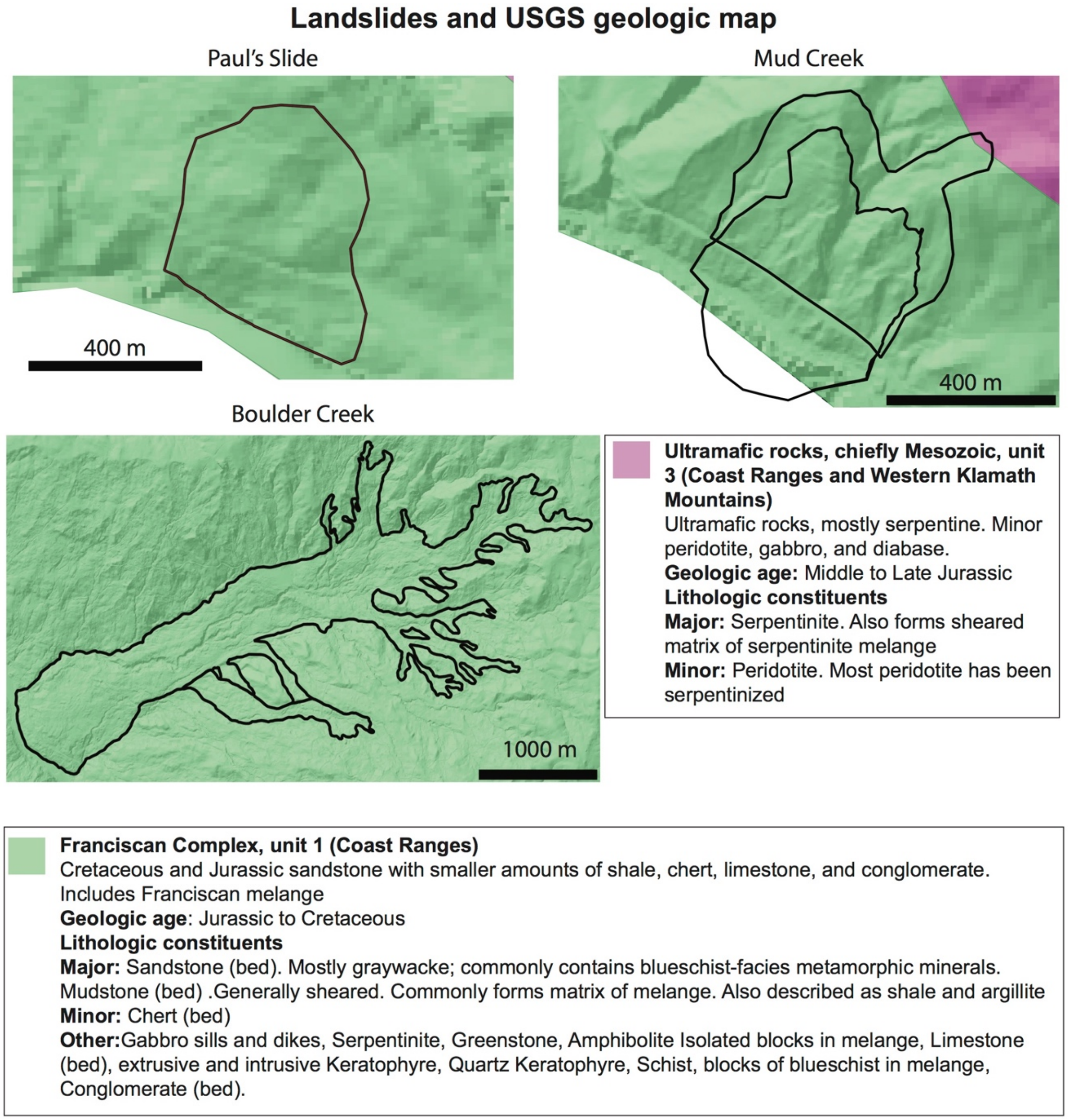

Extended Data Figure 1. Mapped landslides and geologic map. Mapped landslide boundaries and USGS geologic map[28] draped over a hillshade of the topography. Map data: Digital elevation models from USGS (https://earthexplorer.usgs.gov/) and TanDEM-X. TanDEM-X data used is under copyright by the DLR. All rights reserved, used with permission. Geologic map from USGS[28] (https://mrdata.usgs.gov/geology/state/). Software: QGIS Geographic Information System. Open Source Geospatial Foundation Project. http://qgis.osgeo.org.

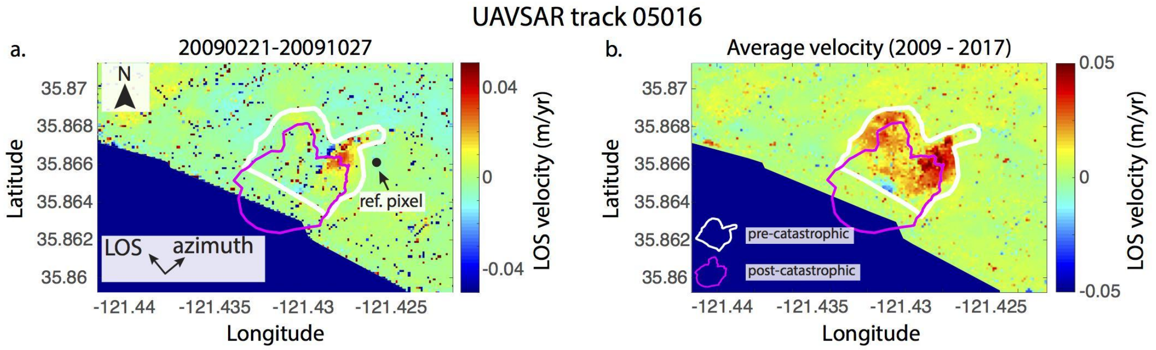

Extended Data Figure 2. UAVSAR interferograms of the Mud Creek landslide. **a**, Example interferogram between February 21, 2009 and October 27, 2009. This interferogram shows the first observed landslide deformation in our data set. Reference pixel corresponds to a stable area outside the landslide. Aircraft line-of-sight (LOS) and azimuth direction are shown with black arrows. **b**, Average velocity Feb. 21, 2009 and Oct. 27, 2016. Negative LOS velocity corresponds to motion towards the aircraft LOS. Solid white and magenta polygons show pre- and post-catastrophic failure boundaries. Software: MATLAB software package R2016b (https://www.mathworks.com/products/matlab.html).

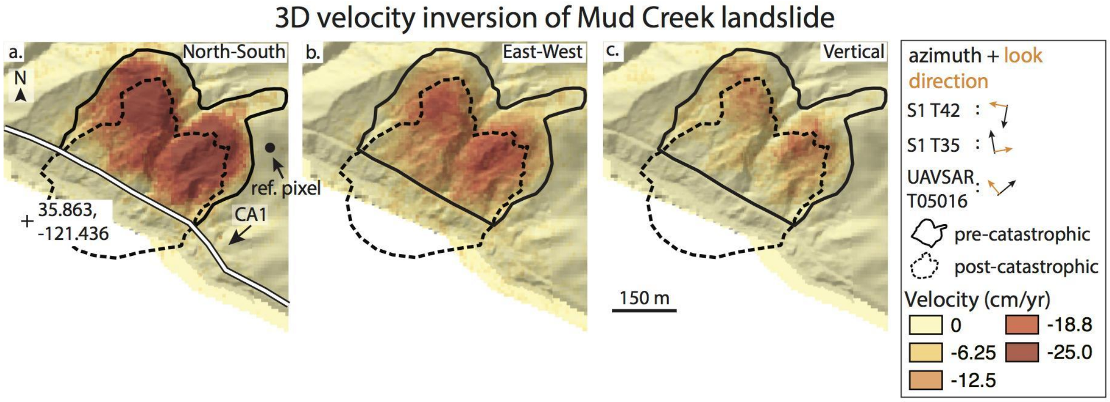

Extended Data Figure 3. 3D velocity maps of the Mud Creek landslide. The average velocity is calculated between February 21, 2009 and May 13, 2017 from Sentinel-1 and UAVSAR and converted to 3D motion and then draped over a lidar hillshade of the topography. Negative velocity values correspond to **a,** South, **b,** West, and **c,** downward motion, respectively. Solid black and dashed black polygons show pre- and post-catastrophic failure boundaries. California Highway 1 shown with black and white line in **a**. The azimuth and look directions of the Sentinel-1A/B and UAVSAR SAR instruments are shown with black and orange arrows in the legend. Reference pixel in **a** corresponds to a stable area outside the landslide. Map data: Digital elevation models from USGS (https://earthexplorer.usgs.gov/) and TanDEM-X. TanDEM-X data used is under copyright by the DLR. All rights reserved, used with permission. Software: QGIS Geographic Information System. Open Source Geospatial Foundation Project. http://qgis.osgeo.org.

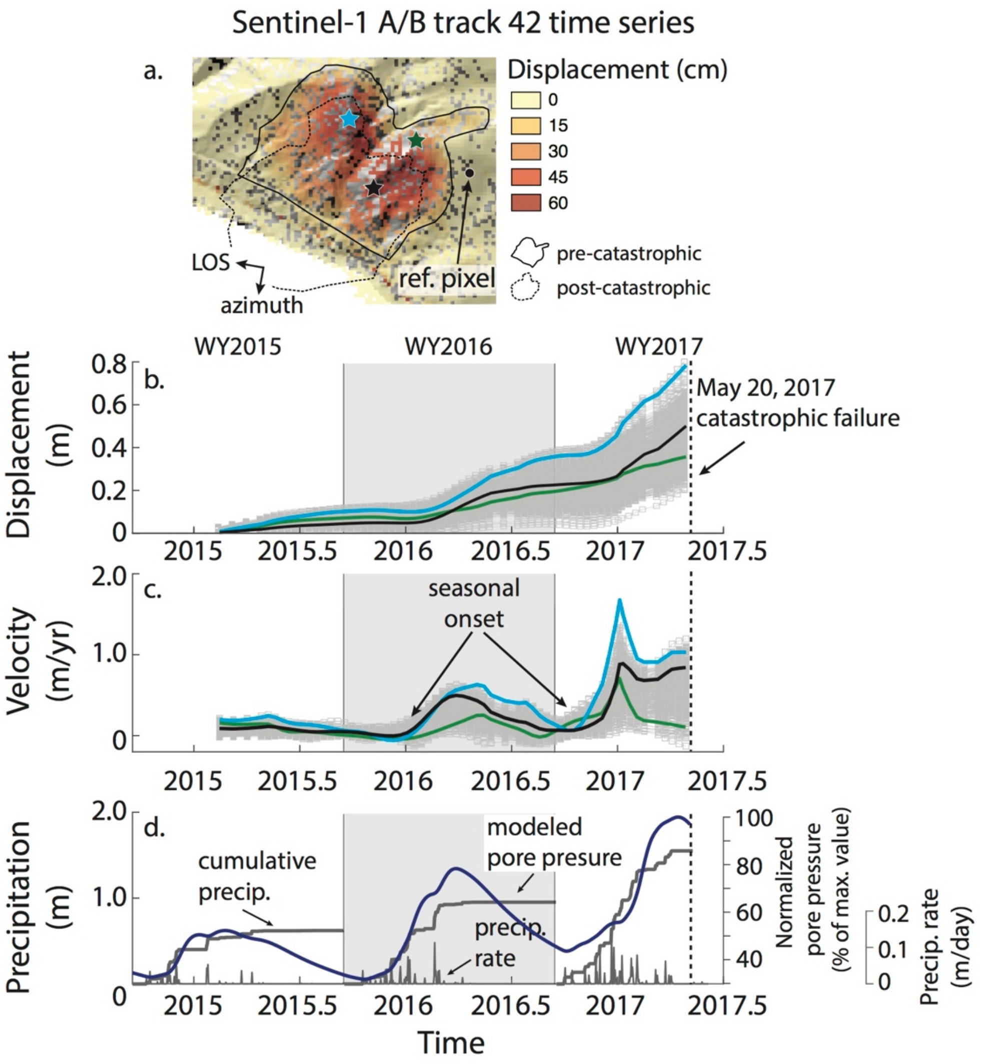

Extended Data Figure 4. **Full spatial time series of the Mud Creek landslide**. The data are from Sentinel-1 A/B descending track 42 and projected onto the downslope direction. Pixels with coherence < 0.2 are masked out. **a**, Cumulative displacement map. Reference pixel corresponds to a stable area outside the landslide. Satellite line-of-sight (LOS) and azimuth direction are shown with black arrows. **b-c**, Displacement and velocity time series. Blue, green, and black lines show the time series for the blue, green, and black stars located in **a**. **d**, Cumulative water-year precipitation, precipitation rate, and modelled pore pressure time series. The normalized pore pressure is defined as the pore pressure divided by the maximum value over the study period. Water years (Oct. 1 – Sep. 30) are highlighted by white and grey boxes. Vertical dashed line shows the date of catastrophic failure. Map data: Digital elevation models from USGS (https://earthexplorer.usgs.gov/) and TanDEM-X. TanDEM-X data used is under copyright by the DLR. All rights reserved, used with permission. Software: QGIS Geographic Information System. Open Source Geospatial Foundation Project. http://qgis.osgeo.org.

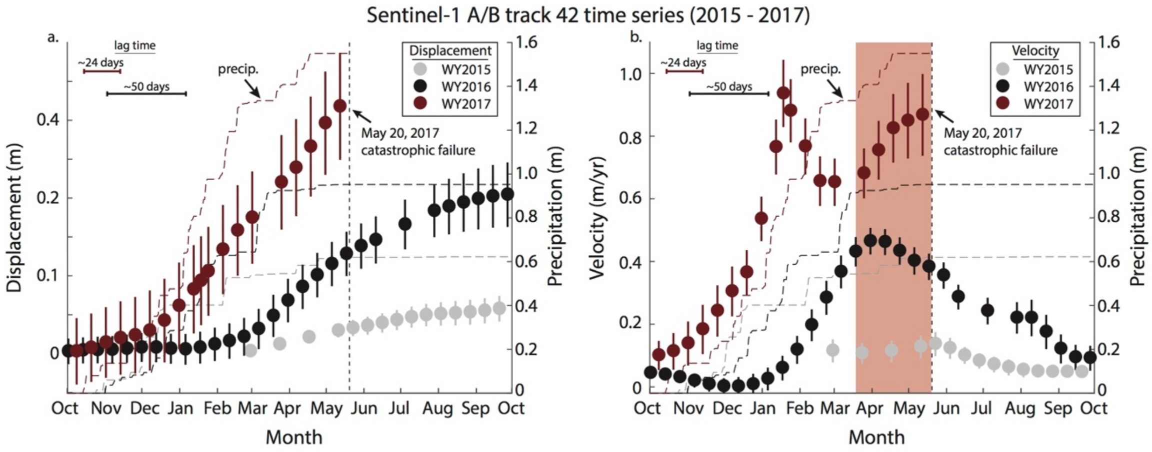

Extended Data Figure 5. Displacement and velocity time series of Mud Creek landslide. a, Downslope displacement and precipitation time series during WY2015-WY2017 condensed into a single calendar year for a representative area (averaged over 60 x 60 m, shown by black star and box in **Fig. 3a**. **b**, Downslope velocity and precipitation time series during WY2015-WY2017 condensed into a single calendar year for a representative area (averaged over 60 x 60 m, shown by black star and box in **Fig. 3a**). Error bars show the standard deviation within the black box shown in **Fig. 3a**. Lag time corresponds to the time between the onset of precipitation and onset of acceleration.

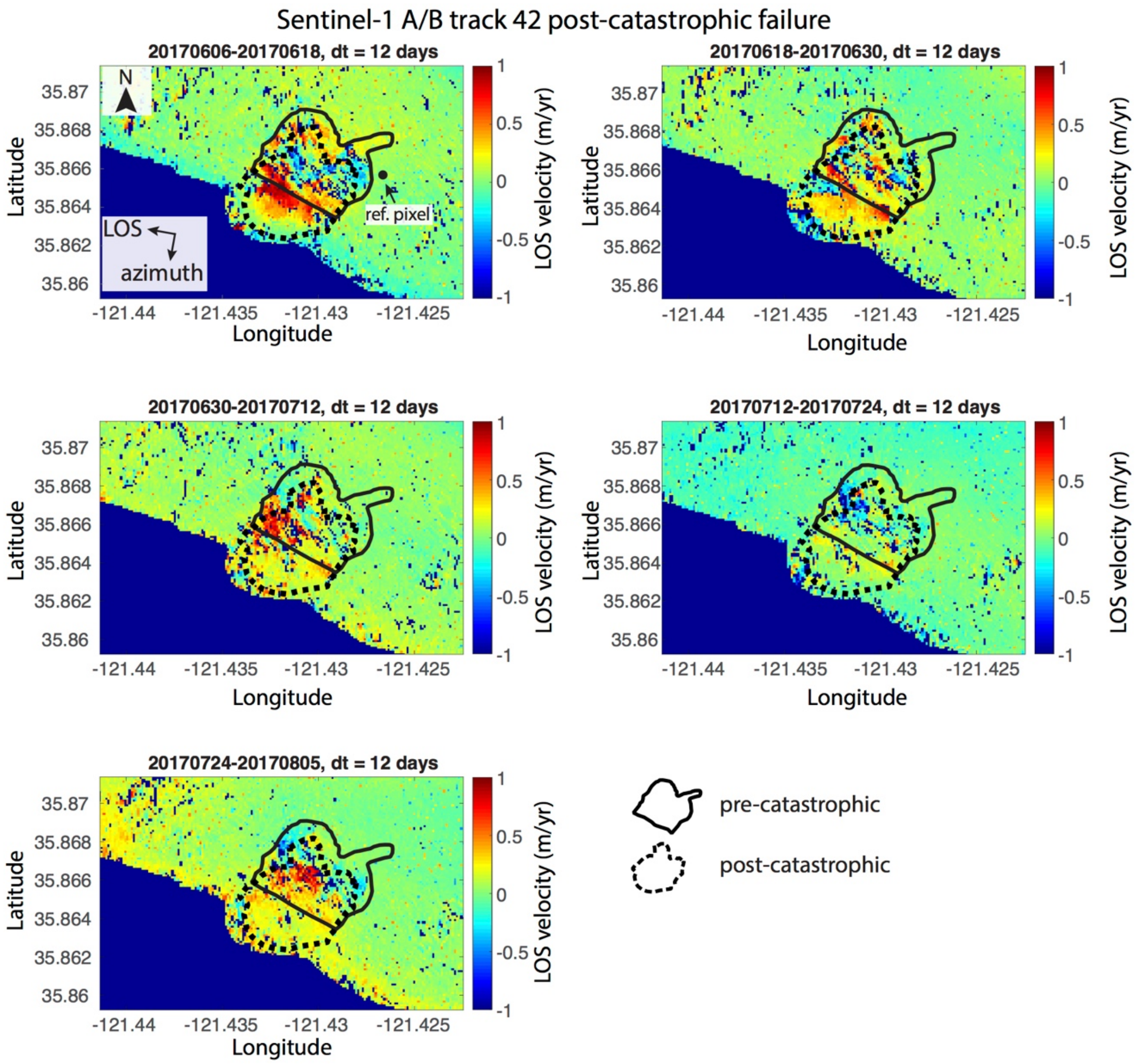

Extended Data Figure 6. Post-catastrophic failure interferograms. Solid black and dashed black polygons show pre- and post-catastrophic failure boundaries. These images show that the landslide failure scar and deposit have continued to deform from natural settlement and human impact (i.e. road construction) for at least two months after the catastrophic failure. Negative line-of-sight (LOS) velocity corresponds to motion towards the satellite LOS. Reference pixel corresponds to a stable area outside the landslide. Satellite (LOS) and azimuth direction are shown with black arrows. Software: MATLAB software package R2016b (https://www.mathworks.com/products/matlab.html).

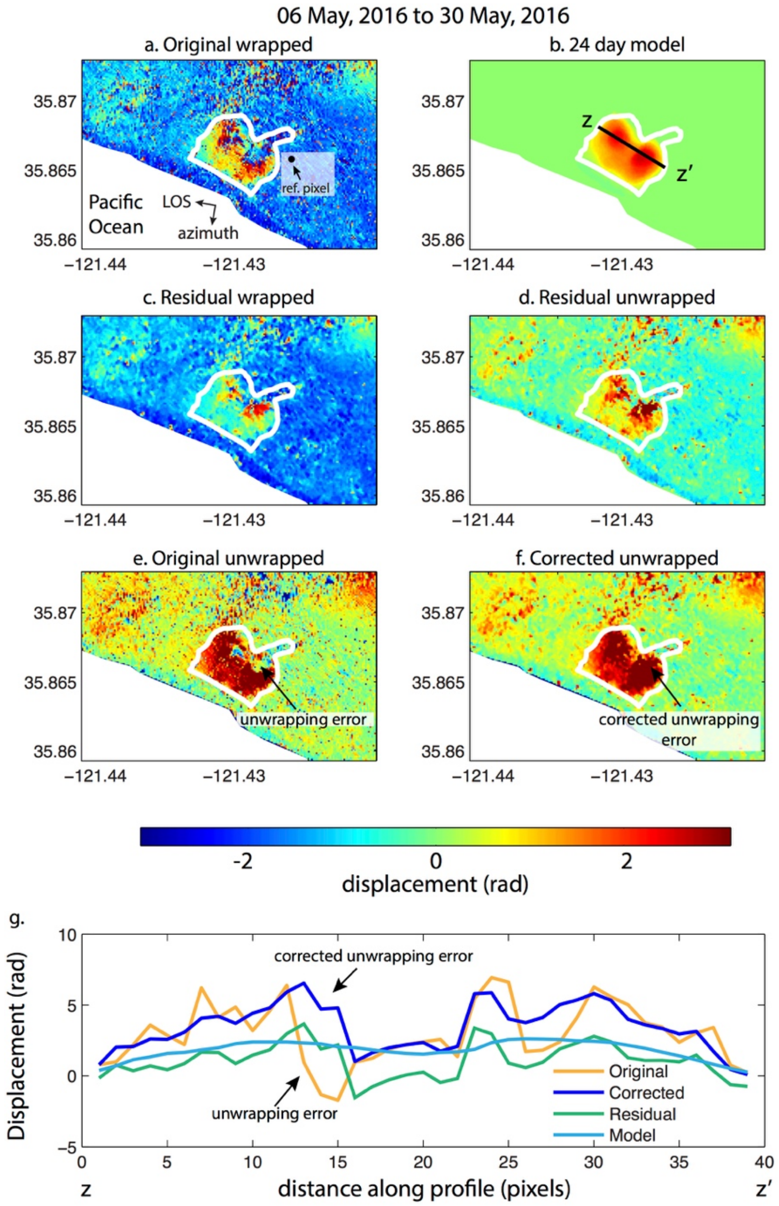

Extended Data Figure 7. Example of unwrapping error correction. Data from Sentinel-1A/B descending track 42. **a**, Original wrapped interferogram. Negative line-of-sight (LOS) velocity corresponds to motion towards the satellite LOS. Reference pixel corresponds to a stable area outside the landslide. Satellite LOS and azimuth direction are shown with black arrows. **b**, 24-day deformation model. **c**, Residual wrapped interferogram (i.e. original interferogram minus the deformation model). **d**, Residual unwrapped. **e**, Original unwrapped interferogram with unwrapping error. **f**, Corrected unwrapped interferogram. **g**, Profiles plotted from z to z' shown in **b**. Solid white polygons show pre-catastrophic failure boundaries. Reference pixel corresponds to a stable area outside the landslide. Software: MATLAB software package R2016b (https://www.mathworks.com/products/matlab.html).

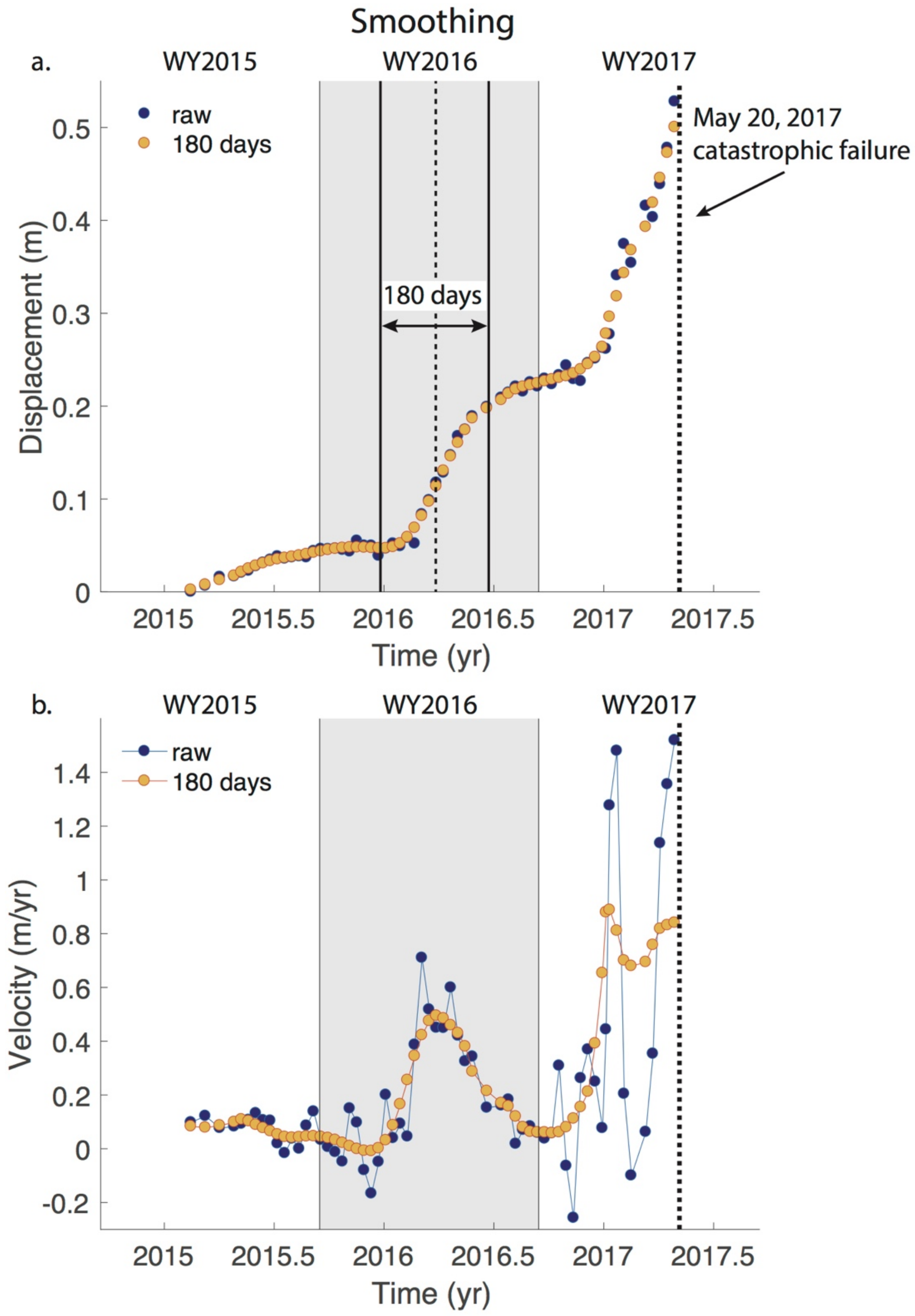

Extended Data Figure 8. Raw and smoothed displacement and velocity time series for the Mud Creek landslide. Data from Sentinel-1A/B descending track 42. The displacement time series is smoothed using a loess filter (locally weighted) with a total window size of 180 days. We find this improves the signal-to-noise ratio and highlights the overall seasonal velocity patterns. Water years (Oct. 1 – Sep. 30) are highlighted by white and grey boxes. Vertical dashed line shows the date of catastrophic failure.

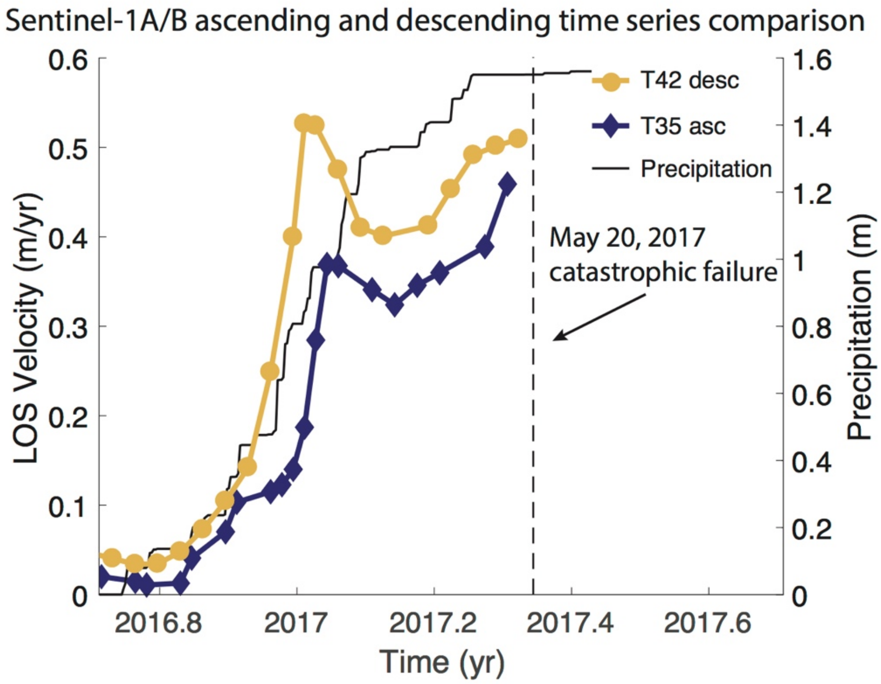

Extended Data Figure 9. Ascending and descending LOS velocity time series comparison for Mud Creek landslide during WY2017. Dashed vertical line shows May 20, 2017, the date of catastrophic failure. The time series comparison shows good agreement. Notably, ascending track 35 had an acquisition one day prior to the catastrophic failure, however the landslide had moved up to several meters on that day[37] and therefore we did not include that data in our analysis. Plotted data corresponds to representative area shown by black star in Figure S3.

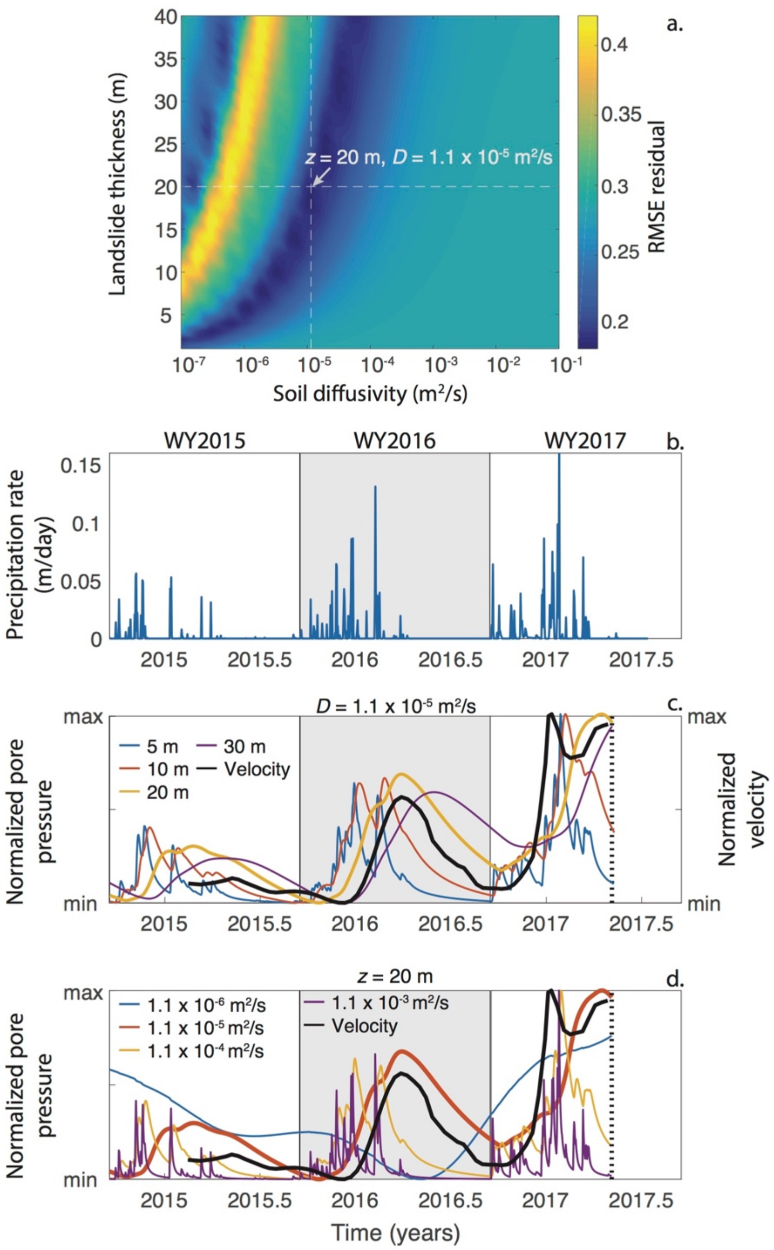

Extended Data Figure 10. Pore pressure diffusion model parameter space. **a,** RMSE residual for velocity and modelled pore pressure during WY2016. For a fixed thickness of 20 m, we find that a diffusivity of 1.1 x 10^{-5} m^2/s minimizes the residual. **b,** Precipitation rate time series. **c**, Modelled pore pressures calculated with a diffusivity of 1.1 x 10^{-5} m^2/s and range of landslide thicknesses. **d,** Modelled pore pressures calculated with a landslide thickness of 20 m and a range of diffusivities. Velocity and pore pressure values are normalized to range between minimum and maximum over the study period. Normalized landslide velocity for the representative area shown by the black star in Figure S3. Data from Sentinel-1A/B descending track 42. Vertical dashed line shows the date of catastrophic failure. Water years (Oct. 1 – Sep. 30) are highlighted by white and grey boxes

Extended Data Table 1. Landslide metrics. Area, length, aspect (i.e. average downslope direction), and slope angle are measured using the TanDEM-X DEM. Length corresponds to the distance from the top to the bottom of the landslide. Average width is calculated as Area/Length. Estimated thickness for Mud Creek is calculated as the estimated volume[36] divided by the area. Estimated thickness of Boulder Creek is from ref 11. Temporally averaged downslope velocities are calculated from the Sentinel-1 A/B data presented in Figure 4.

**Landslide**	**Area (m^2)**	**Length (m)**	**Average Width (m)**	**Estimated Thickness (m)**	**Estimated Volume (m^3)**	**Slope Angle (±1std)**	**Landslide aspect (deg)**	**Average downslope velocity (m/yr)**
Mud Creek (pre-catastrophic failure)	227513	477	477	15-30		32 ± 8.5	203	0.26
Mud Creek (catastrophic failure)	79389	287	277		2.00E6			
Paul's Slide	251628	519	485			25 ± 11	192	0.17
Boulder Creek	3.11E6	4850	641	40		15 ± 8	240	0.47

Supplementary Table 1. SAR data used from Sentinel-1 A/B descending track 42 processing of Mud Creek landslide. Columns correspond to the interferogram number, acquisition date of the master image, acquisition date of the slave image, the change in time between master and slave images, information on whether interferogram was used to construct the deformation, and information on whether the deformation model was used during processing. Red text shows information for post-catastrophic failure interferograms.

Supplementary Table 2. SAR data used from Sentinel-1 A/B descending track 35 processing of Mud Creek landslide. Columns correspond to the interferogram number, acquisition date of the master image, acquisition date of the slave image, the change in time between master and slave images, information on whether interferogram was used to construct the deformation, and information on whether the deformation model was used during processing.

Supplementary Table 3. SAR data used from Sentinel-1 A/B descending track 42 processing of Paul's Slide. Columns correspond to the interferogram number, acquisition date of the master image, acquisition date of the slave image, the change in time between master and slave images, and information on whether the deformation model was used during processing.

Supplementary Table 4. SAR data used from Sentinel-1 A/B descending track 35 processing of Boulder Creek landslide. Columns correspond to the interferogram number, acquisition date of the master image, acquisition date of the slave image, the change in time between master and slave images, information on whether interferogram was used to construct the deformation, and information on whether the deformation model was used during processing.

Supplementary Table 5. SAR data used from UAVSAR track 05016 processing of Mud Creek landslide. Columns correspond to the interferogram number, acquisition date of the master image, acquisition date of the slave image, the change in time between master and slave images, and information on whether the deformation model was used during processing.

Supplementary Movie 1. Velocity time series movie for Mud Creek landslide. Data from Sentinel-1 A/B descending track 42 and projected onto the downslope direction. (Top) Velocity time series map. White polygon shows pre-catastrophic failure boundaries and red polygon shows post-catastrophic failure boundaries. SAR acquisition date shown in title. (Bottom) Velocity (orange) and precipitation (blue) time series. Velocity time series corresponds to the black circle shown on velocity map.
